# Poly[di-μ_3_-chlorido-[μ_2_-(3-pyrid­yl)(4-pyrid­yl)methanone-κ^2^ *N*:*N*′]dicopper(I)]

**DOI:** 10.1107/S1600536808038385

**Published:** 2008-11-22

**Authors:** Jennifer L. Hittle, Robert L. LaDuca

**Affiliations:** a Lyman Briggs College, Department of Chemistry, Michigan State University, East Lansing, MI 48825, USA

## Abstract

In the title compound, [Cu_2_Cl_2_(C_11_H_8_N_2_O)]_*n*_, stair-like ribbons of formula [Cu_2_Cl_2_]_*n*_ are linked into coordination polymer layers by tethering (3-pyrid­yl)(4-pyrid­yl)methanone (3,4′-dpk) ligands. The two distinct Cu^I^ centres both adopt distorted CuNCl_3_ tetra­hedral coordinations. Individual [Cu_2_Cl_2_(3,4′-dpk)]_*n*_ layers stack in an *AB* pattern along the *c* direction by way of weak C—H⋯O inter­actions between the pyridyl rings and ketone O atoms.

## Related literature

For copper molybdate coordination polymers with (3-pyridyl)(4-pyridyl)­methanone and the synthesis of this ligand, see: Montney & LaDuca (2008 ▶). For data-handling software, see: Sheldrick (2003 ▶).
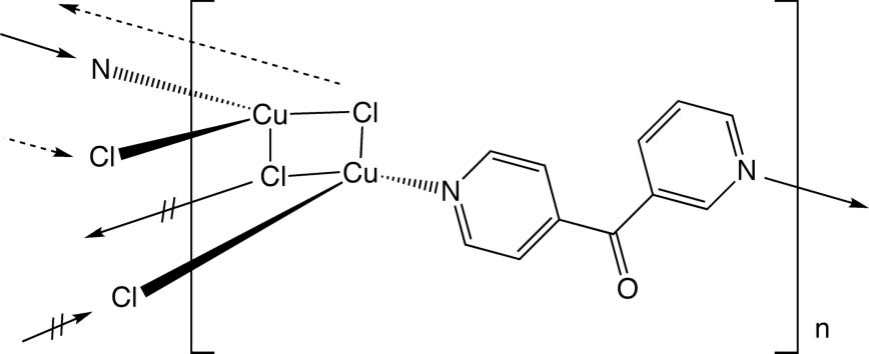


## Experimental

### 

#### Crystal data

[Cu_2_Cl_2_(C_11_H_8_N_2_O)]
*M* *_r_* = 382.17
Monoclinic, 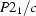

*a* = 3.7765 (7) Å
*b* = 25.935 (5) Å
*c* = 12.339 (2) Å
β = 94.462 (3)°
*V* = 1204.9 (4) Å^3^
*Z* = 4
Mo *K*α radiation
μ = 3.96 mm^−1^
*T* = 173 (2) K
0.22 × 0.14 × 0.08 mm

#### Data collection

Bruker SMART 1K CCD diffractometer
Absorption correction: multi-scan (TWINABS; Sheldrick, 2007 ▶) *T*_min_ = 0.503, *T*_max_ = 0.731
20010 measured reflections
2794 independent reflections
2435 reflections with *I* > 2σ(*I*)
*R*_int_ = 0.042

#### Refinement

*R*[*F* ^2^ > 2σ(*F* ^2^)] = 0.039
*wR*(*F* ^2^) = 0.095
*S* = 1.06
2794 reflections
163 parameters
H-atom parameters constrained
Δρ_max_ = 0.97 e Å^−3^
Δρ_min_ = −0.48 e Å^−3^

### 

Data collection: *SMART* (Bruker, 2003 ▶); cell refinement: *SAINT-Plus* (Bruker, 2003 ▶); data reduction: *SAINT-Plus*; program(s) used to solve structure: *SHELXS97* (Sheldrick, 2008 ▶); program(s) used to refine structure: *SHELXL97* (Sheldrick, 2008 ▶); molecular graphics: *CrystalMaker* (Palmer, 2007 ▶); software used to prepare material for publication: *SHELXL97*.

## Supplementary Material

Crystal structure: contains datablocks I, global. DOI: 10.1107/S1600536808038385/hb2839sup1.cif

Structure factors: contains datablocks I. DOI: 10.1107/S1600536808038385/hb2839Isup2.hkl

Additional supplementary materials:  crystallographic information; 3D view; checkCIF report

## Figures and Tables

**Table 1:** Selected bond lengths (Å)

Cu1—N1	1.995 (3)
Cu1—Cl2	2.2787 (10)
Cu1—Cl2^i^	2.4081 (10)
Cu1—Cl1	2.5854 (11)
Cu2—N2^ii^	2.002 (3)
Cu2—Cl1	2.3173 (10)
Cu2—Cl1^i^	2.4118 (10)
Cu2—Cl2^i^	2.5343 (11)

Symmetry codes: (i) 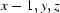; (ii) 
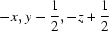
.

**Table 2:** Hydrogen-bond geometry (Å, °)

*D*—H⋯*A*	*D*—H	H⋯*A*	*D*⋯*A*	*D*—H⋯*A*
C5—H5⋯O1^iii^	0.93	2.35	3.112 (5)	139

Symmetry code: (iii) 
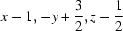
.

**Fig. 1.**
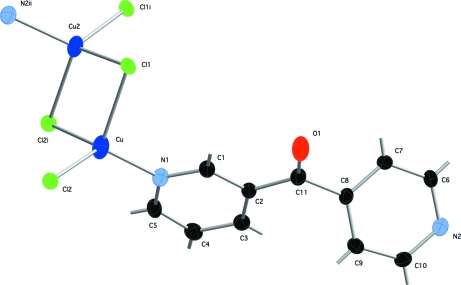
Asymmetric unit of (I), expanded to show the metal coordination spheres, showing 50% probability ellipsoids. Hydrogen atom positions are shown as gray sticks. Symmetry codes: (1) x - 1, y, z (ii) -x, y - 1/2, -z + 1/2

**Fig. 2.**
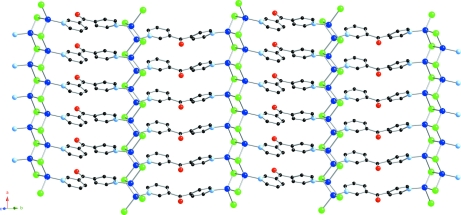
A single [Cu2Cl2(3,4'-dpk)]n layer in (I) formed by the linkage of [Cu2Cl2]n ladders through the diimine ligands.

**Fig. 3.**
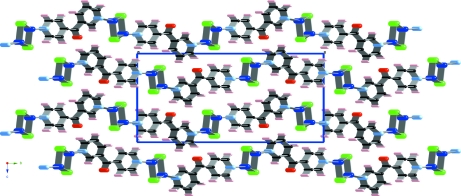
Packing diagram illustrating the AB layer stacking pattern, which forms the 3-D crystal structure of (I) through weak C—H···O interactions between pyridyl rings and ketone O atoms.

## supplementary crystallographic information

### Comment

The kinked-donor disposed dipodal tethering ligand (3-pyridyl)(4-pyridyl)methanone (3,4'-dpk) has been rarely utilized for the construction of coordination polymer solids. Two copper molybdate phases incoporating this ligand have been reported recently (Montney & LaDuca, 2008). In an attempt to extend this chemistry into dicarboxylate systems, yellow crystals of the title compound, (I), were obtained.

The asymmetric unit of (I) contains two monovalent copper atoms, two chloride ions and one complete 3,4'-dpk ligand (Fig. 1). The coordination environment at each Cu atom is a distorted {CuCl_3_N} tetrahedron (Table 1). The Cu and Cl atoms link into [Cu_2_Cl_2_]_n_ stair-like ribbons that are oriented parallel to the *a* crystal direction. The Cu···Cu distances across the `steps' of the stair-like ribbons measure 2.857 (1) Å and 3.153 (1) Å, respectively.

Parallel [Cu_2_Cl_2_]_n_ ribbons are covalently connected into [Cu_2_Cl_2_(3,4'-dpk)]_n_ coordination polymer layers, arranged parallel to the *ab* crystal planes, *via* the tethering 3,4'-dpk ligands (Fig. 2). The Cu···Cu contact distances across the diimine ligands measure 11.573 (3) Å. The dihedral angle between the pyridyl rings within a 3,4'-dpk ligand is 46.53 (17)°. Individual [Cu_2_Cl_2_(3,4'-dpk)]_n_ layers stack in an *AB* pattern along the *c* crystal direction through weak C—H···O supramolecular interactions between the pyridyl rings and ketone O atoms (Fig. 3), with a C···O contact distance of 3.112 (5) Å (Table 2).

### Experimental

All chemicals were obtained commercially with the exception of (3-pyridyl)(4-pyridyl)methanone (Montney & LaDuca, 2008). A mixture of copper(II) chloride dihydrate (63 mg, 0.37 mmol), phthalic acid (61 mg, 0.37 mmol), (3-pyridyl)(4-pyridyl)methanone (136 mg, 0.74 mmol) and 10.0 g water (555 mmol) was placed in a 23 ml Teflon-lined Parr acid digestion bomb, which was then heated under autogenous pressure at 393 K for 48 h. Yellow–orange blocks of (I) were obtained.

### Refinement

Reflection data were collected on a non-merohedrally twinned crystal. The twin law was determined with *CELL-NOW* (Sheldrick, 2003). The structure was solved and refined using reflections from only the major twin component, whose reflection file was generated using *TWINABS* (Sheldrick, 2007). All H atoms bound to C atoms were placed in calculated positions, with C—H = 0.95 Å and refined in riding mode with *U*_iso_ = 1.2*U*_eq_(C).

### Crystal data

**Table:**

[Cu_2_Cl_2_(C_11_H_8_N_2_O)]	*F*_000_ = 752
*M**_r_* = 382.17	*D*_x_ = 2.107 Mg m^−^^3^
Monoclinic, *P*2_1_/*c*	Mo *K*α radiation λ = 0.71073 Å
Hall symbol: -P 2ybc	Cell parameters from 20010 reflections
*a* = 3.7765 (7) Å	θ = 1.6–28.3º
*b* = 25.935 (5) Å	µ = 3.96 mm^−^^1^
*c* = 12.339 (2) Å	*T* = 173 (2) K
β = 94.462 (3)º	Block, yellow
*V* = 1204.9 (4) Å^3^	0.22 × 0.14 × 0.08 mm
*Z* = 4	

### Data collection

**Table:**

Bruker SMART 1K CCD diffractometer	2794 independent reflections
Radiation source: fine-focus sealed tube	2435 reflections with *I* > 2σ(*I*)
Monochromator: graphite	*R*_int_ = 0.042
*T* = 173(2) K	θ_max_ = 28.3º
ω scans	θ_min_ = 1.6º
Absorption correction: multi-scan(TWINABS; Sheldrick, 2007)	*h* = −5→4
*T*_min_ = 0.503, *T*_max_ = 0.731	*k* = 0→34
20010 measured reflections	*l* = 0→16

### Refinement

**Table:**

Refinement on *F*^2^	Secondary atom site location: difference Fourier map
Least-squares matrix: full	Hydrogen site location: inferred from neighbouring sites
*R*[*F*^2^ > 2σ(*F*^2^)] = 0.039	H-atom parameters constrained
*wR*(*F*^2^) = 0.095	*w* = 1/[σ^2^(*F*_o_^2^) + (0.0339*P*)^2^ + 4.4918*P*] where *P* = (*F*_o_^2^ + 2*F*_c_^2^)/3
*S* = 1.06	(Δ/σ)_max_ = 0.001
2794 reflections	Δρ_max_ = 0.97 e Å^−^^3^
163 parameters	Δρ_min_ = −0.48 e Å^−^^3^
Primary atom site location: structure-invariant direct methods	Extinction correction: none

### Special details

**Table:**

Geometry. All e.s.d.'s (except the e.s.d. in the dihedral angle between two l.s. planes) are estimated using the full covariance matrix. The cell e.s.d.'s are taken into account individually in the estimation of e.s.d.'s in distances, angles and torsion angles; correlations between e.s.d.'s in cell parameters are only used when they are defined by crystal symmetry. An approximate (isotropic) treatment of cell e.s.d.'s is used for estimating e.s.d.'s involving l.s. planes.
Refinement. Refinement of *F*^2^ against ALL reflections for the major twin component. The weighted *R*-factor *wR* and goodness of fit *S* are based on *F*^2^, conventional *R*-factors *R* are based on *F*, with *F* set to zero for negative *F*^2^. The threshold expression of *F*^2^ > σ(*F*^2^) is used only for calculating *R*-factors(gt) *etc*. and is not relevant to the choice of reflections for refinement. *R*-factors based on *F*^2^ are statistically about twice as large as those based on *F*, and *R*- factors based on ALL data will be even larger.

### Fractional atomic coordinates and isotropic or equivalent isotropic displacement parameters (Å^2^)

**Table:**

	*x*	*y*	*z*	*U*_iso_*/*U*_eq_	
Cu1	0.30817 (14)	0.633401 (18)	0.12987 (4)	0.03284 (15)	
Cu2	−0.09246 (14)	0.576023 (18)	0.27566 (4)	0.03282 (15)	
Cl1	0.4078 (2)	0.62217 (3)	0.33820 (6)	0.01995 (17)	
Cl2	0.7724 (2)	0.58732 (3)	0.07265 (7)	0.02118 (18)	
N1	0.1879 (8)	0.70719 (11)	0.0999 (2)	0.0217 (6)	
C2	0.2012 (9)	0.79762 (13)	0.1460 (3)	0.0186 (6)	
N2	0.1356 (8)	0.99919 (11)	0.2175 (2)	0.0228 (6)	
C8	0.2454 (9)	0.89246 (12)	0.2191 (3)	0.0193 (7)	
C7	0.1332 (10)	0.91779 (14)	0.3097 (3)	0.0237 (7)	
H7	0.0973	0.8995	0.3728	0.028*	
C1	0.2845 (9)	0.74576 (13)	0.1677 (3)	0.0205 (7)	
H1	0.4139	0.7378	0.2327	0.025*	
O1	0.4939 (9)	0.82138 (10)	0.3137 (2)	0.0371 (7)	
C9	0.2995 (9)	0.92152 (14)	0.1272 (3)	0.0224 (7)	
H9	0.3696	0.9058	0.0646	0.027*	
C5	−0.0002 (10)	0.71977 (14)	0.0055 (3)	0.0238 (7)	
H5	−0.0729	0.6934	−0.0423	0.029*	
C4	−0.0885 (10)	0.76977 (14)	−0.0230 (3)	0.0236 (7)	
H4	−0.2124	0.7768	−0.0895	0.028*	
C3	0.0094 (9)	0.80956 (13)	0.0486 (3)	0.0210 (7)	
H3	−0.0523	0.8435	0.0316	0.025*	
C10	0.2469 (9)	0.97448 (13)	0.1308 (3)	0.0216 (7)	
H10	0.2911	0.9939	0.0699	0.026*	
C6	0.0756 (10)	0.97045 (14)	0.3052 (3)	0.0252 (7)	
H6	−0.0083	0.9867	0.3653	0.030*	
C11	0.3264 (10)	0.83580 (13)	0.2312 (3)	0.0218 (7)	

### Atomic displacement parameters (Å^2^)

**Table:**

	*U*^11^	*U*^22^	*U*^33^	*U*^12^	*U*^13^	*U*^23^
Cu1	0.0365 (3)	0.0196 (2)	0.0417 (3)	0.00451 (19)	−0.0013 (2)	−0.0024 (2)
Cu2	0.0436 (3)	0.0188 (2)	0.0359 (3)	−0.0030 (2)	0.0024 (2)	−0.00250 (19)
Cl1	0.0204 (4)	0.0227 (4)	0.0165 (4)	−0.0004 (3)	−0.0001 (3)	−0.0049 (3)
Cl2	0.0234 (4)	0.0203 (4)	0.0197 (4)	0.0012 (3)	0.0005 (3)	−0.0002 (3)
N1	0.0223 (15)	0.0191 (14)	0.0232 (15)	0.0003 (11)	−0.0024 (11)	0.0000 (11)
C2	0.0198 (16)	0.0178 (15)	0.0181 (15)	0.0006 (12)	0.0008 (12)	−0.0004 (12)
N2	0.0243 (15)	0.0187 (14)	0.0251 (15)	−0.0012 (11)	0.0001 (12)	−0.0002 (12)
C8	0.0227 (17)	0.0155 (15)	0.0191 (16)	−0.0016 (12)	−0.0020 (13)	−0.0007 (12)
C7	0.035 (2)	0.0209 (17)	0.0149 (16)	−0.0059 (14)	0.0029 (14)	−0.0002 (13)
C1	0.0234 (17)	0.0195 (16)	0.0177 (16)	0.0010 (13)	−0.0035 (13)	0.0006 (13)
O1	0.0581 (19)	0.0229 (13)	0.0270 (14)	0.0042 (13)	−0.0178 (13)	−0.0015 (11)
C9	0.0263 (18)	0.0235 (17)	0.0174 (16)	0.0006 (14)	0.0021 (13)	−0.0013 (13)
C5	0.0278 (18)	0.0210 (16)	0.0219 (17)	−0.0008 (14)	−0.0021 (14)	−0.0069 (14)
C4	0.0273 (19)	0.0265 (18)	0.0162 (16)	0.0018 (14)	−0.0040 (13)	0.0006 (13)
C3	0.0235 (17)	0.0200 (16)	0.0192 (16)	0.0032 (13)	0.0002 (13)	0.0022 (13)
C10	0.0233 (17)	0.0224 (17)	0.0190 (16)	−0.0014 (13)	0.0009 (13)	0.0043 (13)
C6	0.035 (2)	0.0191 (17)	0.0219 (17)	−0.0034 (14)	0.0056 (15)	−0.0053 (14)
C11	0.0265 (18)	0.0185 (16)	0.0197 (16)	0.0001 (13)	−0.0025 (13)	0.0010 (13)

### Geometric parameters (Å, °)

**Table:**

Cu1—N1	1.995 (3)	C8—C9	1.390 (5)
Cu1—Cl2	2.2787 (10)	C8—C7	1.391 (5)
Cu1—Cl2^i^	2.4081 (10)	C8—C11	1.506 (5)
Cu1—Cl1	2.5854 (11)	C7—C6	1.383 (5)
Cu2—N2^ii^	2.002 (3)	C7—H7	0.9300
Cu2—Cl1	2.3173 (10)	C1—H1	0.9300
Cu2—Cl1^i^	2.4118 (10)	O1—C11	1.216 (4)
Cu2—Cl2^i^	2.5343 (11)	C9—C10	1.389 (5)
N1—C1	1.336 (4)	C9—H9	0.9300
N1—C5	1.356 (5)	C5—C4	1.378 (5)
C2—C3	1.389 (5)	C5—H5	0.9300
C2—C1	1.403 (5)	C4—C3	1.390 (5)
C2—C11	1.494 (5)	C4—H4	0.9300
N2—C10	1.343 (5)	C3—H3	0.9300
N2—C6	1.348 (5)	C10—H10	0.9300
N2—Cu2^iii^	2.002 (3)	C6—H6	0.9300
			
N1—Cu1—Cl2	127.98 (9)	C10—N2—Cu2^iii^	122.7 (2)
N1—Cu1—Cl2^i^	104.34 (9)	C6—N2—Cu2^iii^	119.9 (2)
Cl2—Cu1—Cl2^i^	107.34 (4)	C9—C8—C7	118.2 (3)
N1—Cu1—Cl1	107.80 (9)	C9—C8—C11	124.6 (3)
Cl2—Cu1—Cl1	101.13 (3)	C7—C8—C11	117.0 (3)
Cl2^i^—Cu1—Cl1	106.83 (3)	C6—C7—C8	119.4 (3)
N1—Cu1—Cu2	119.49 (9)	C6—C7—H7	120.3
Cl2—Cu1—Cu2	112.32 (3)	C8—C7—H7	120.3
Cl2^i^—Cu1—Cu2	56.78 (3)	N1—C1—C2	123.5 (3)
Cl1—Cu1—Cu2	50.09 (2)	N1—C1—H1	118.2
N2^ii^—Cu2—Cl1	124.54 (9)	C2—C1—H1	118.2
N2^ii^—Cu2—Cl1^i^	114.36 (9)	C10—C9—C8	118.7 (3)
Cl1—Cu2—Cl1^i^	105.97 (4)	C10—C9—H9	120.7
N2^ii^—Cu2—Cl2^i^	98.38 (9)	C8—C9—H9	120.7
Cl1—Cu2—Cl2^i^	111.46 (3)	N1—C5—C4	123.1 (3)
Cl1^i^—Cu2—Cl2^i^	99.00 (3)	N1—C5—H5	118.5
N2^ii^—Cu2—Cu1	126.42 (9)	C4—C5—H5	118.5
Cl1—Cu2—Cu1	58.85 (3)	C5—C4—C3	119.3 (3)
Cl1^i^—Cu2—Cu1	114.14 (3)	C5—C4—H4	120.3
Cl2^i^—Cu2—Cu1	52.64 (3)	C3—C4—H4	120.3
Cu2—Cl1—Cu2^iv^	105.97 (4)	C2—C3—C4	118.7 (3)
Cu2—Cl1—Cu1	71.05 (3)	C2—C3—H3	120.7
Cu2^iv^—Cl1—Cu1	78.15 (3)	C4—C3—H3	120.7
Cu1—Cl2—Cu1^iv^	107.34 (4)	N2—C10—C9	123.5 (3)
Cu1—Cl2—Cu2^iv^	81.67 (3)	N2—C10—H10	118.3
Cu1^iv^—Cl2—Cu2^iv^	70.58 (3)	C9—C10—H10	118.3
C1—N1—C5	117.2 (3)	N2—C6—C7	122.9 (3)
C1—N1—Cu1	123.7 (2)	N2—C6—H6	118.6
C5—N1—Cu1	119.1 (2)	C7—C6—H6	118.6
C3—C2—C1	118.2 (3)	O1—C11—C2	120.1 (3)
C3—C2—C11	125.2 (3)	O1—C11—C8	118.1 (3)
C1—C2—C11	116.6 (3)	C2—C11—C8	121.8 (3)
C10—N2—C6	117.3 (3)		
			
N1—Cu1—Cu2—N2^ii^	158.68 (15)	Cl2—Cu1—N1—C1	−95.8 (3)
Cl2—Cu1—Cu2—N2^ii^	−26.27 (12)	Cl2^i^—Cu1—N1—C1	138.1 (3)
Cl2^i^—Cu1—Cu2—N2^ii^	70.41 (11)	Cl1—Cu1—N1—C1	24.8 (3)
Cl1—Cu1—Cu2—N2^ii^	−112.18 (12)	Cu2—Cu1—N1—C1	78.4 (3)
N1—Cu1—Cu2—Cl1	−89.14 (10)	Cl2—Cu1—N1—C5	83.7 (3)
Cl2—Cu1—Cu2—Cl1	85.91 (4)	Cl2^i^—Cu1—N1—C5	−42.4 (3)
Cl2^i^—Cu1—Cu2—Cl1	−177.41 (4)	Cl1—Cu1—N1—C5	−155.8 (3)
N1—Cu1—Cu2—Cl1^i^	5.53 (10)	Cu2—Cu1—N1—C5	−102.1 (3)
Cl2—Cu1—Cu2—Cl1^i^	−179.41 (4)	C9—C8—C7—C6	0.8 (5)
Cl2^i^—Cu1—Cu2—Cl1^i^	−82.74 (4)	C11—C8—C7—C6	176.2 (3)
Cl1—Cu1—Cu2—Cl1^i^	94.67 (4)	C5—N1—C1—C2	0.1 (5)
N1—Cu1—Cu2—Cl2^i^	88.27 (10)	Cu1—N1—C1—C2	179.6 (3)
Cl2—Cu1—Cu2—Cl2^i^	−96.67 (4)	C3—C2—C1—N1	−0.3 (5)
Cl1—Cu1—Cu2—Cl2^i^	177.41 (4)	C11—C2—C1—N1	179.3 (3)
N2^ii^—Cu2—Cl1—Cu2^iv^	44.13 (12)	C7—C8—C9—C10	1.4 (5)
Cl1^i^—Cu2—Cl1—Cu2^iv^	180.0	C11—C8—C9—C10	−173.6 (3)
Cl2^i^—Cu2—Cl1—Cu2^iv^	−73.30 (4)	C1—N1—C5—C4	0.9 (6)
Cu1—Cu2—Cl1—Cu2^iv^	−71.09 (3)	Cu1—N1—C5—C4	−178.6 (3)
N2^ii^—Cu2—Cl1—Cu1	115.22 (11)	N1—C5—C4—C3	−1.7 (6)
Cl1^i^—Cu2—Cl1—Cu1	−108.91 (3)	C1—C2—C3—C4	−0.5 (5)
Cl2^i^—Cu2—Cl1—Cu1	−2.21 (3)	C11—C2—C3—C4	179.9 (3)
N1—Cu1—Cl1—Cu2	113.91 (9)	C5—C4—C3—C2	1.5 (5)
Cl2—Cu1—Cl1—Cu2	−109.88 (4)	C6—N2—C10—C9	0.5 (5)
Cl2^i^—Cu1—Cl1—Cu2	2.26 (3)	Cu2^iii^—N2—C10—C9	175.9 (3)
N1—Cu1—Cl1—Cu2^iv^	−134.42 (9)	C8—C9—C10—N2	−2.1 (6)
Cl2—Cu1—Cl1—Cu2^iv^	1.79 (3)	C10—N2—C6—C7	1.8 (6)
Cl2^i^—Cu1—Cl1—Cu2^iv^	113.93 (3)	Cu2^iii^—N2—C6—C7	−173.7 (3)
Cu2—Cu1—Cl1—Cu2^iv^	111.67 (3)	C8—C7—C6—N2	−2.5 (6)
N1—Cu1—Cl2—Cu1^iv^	55.05 (12)	C3—C2—C11—O1	−179.8 (4)
Cl2^i^—Cu1—Cl2—Cu1^iv^	180.0	C1—C2—C11—O1	0.6 (5)
Cl1—Cu1—Cl2—Cu1^iv^	−68.25 (4)	C3—C2—C11—C8	1.3 (5)
Cu2—Cu1—Cl2—Cu1^iv^	−119.49 (3)	C1—C2—C11—C8	−178.3 (3)
N1—Cu1—Cl2—Cu2^iv^	121.61 (11)	C9—C8—C11—O1	131.7 (4)
Cl2^i^—Cu1—Cl2—Cu2^iv^	−113.44 (3)	C7—C8—C11—O1	−43.4 (5)
Cl1—Cu1—Cl2—Cu2^iv^	−1.69 (3)	C9—C8—C11—C2	−49.4 (5)
Cu2—Cu1—Cl2—Cu2^iv^	−52.93 (3)	C7—C8—C11—C2	135.5 (4)

Symmetry codes: (i) *x*−1, *y*, *z*; (ii) −*x*, *y*−1/2, −*z*+1/2; (iii) −*x*, *y*+1/2, −*z*+1/2; (iv) *x*+1, *y*, *z*.

### Hydrogen-bond geometry (Å, °)

**Table:**

*D*—H···*A*	*D*—H	H···*A*	*D*···*A*	*D*—H···*A*
C5—H5···O1^v^	0.93	2.35	3.112 (5)	139

Symmetry codes: (v) *x*−1, −*y*+3/2, *z*−1/2.
